# Experimental Investigations into the Deformation and Failure of Polyurea-Coated Steel Plates Experiencing Localized Air Blast Loads

**DOI:** 10.3390/polym17182481

**Published:** 2025-09-14

**Authors:** Dian Li, Zichun He, Mao Li

**Affiliations:** 1 College of Naval Architecture and Ocean Engineering, Naval University of Engineering, Wuhan 430033, China; 2 Naval Academy of Research, Beijing 100161, China

**Keywords:** mechanics of explosion, air blast shock wave, polyurea coating, composite structure, experimental investigation

## Abstract

Experimental investigations were conducted on the polyurea-coated steel plates experiencing localized air blast loads. The blast distance and polyurea coating position on the deformation and failure modes of the polyurea-coated steel plates were compared with those of homogeneous steel plates. The dynamic response failure and stress wave propagation characteristics of the polyurea-coated steel plates were studied. The results showed that under the localized air blast loads, the polyurea-coated steel plates demonstrated three failure modes: large flexural deformation, flexural deformation plus plugging breach, and flexural deformation plus plugging breach plus petal-like cracking. Under the same blast distance and surface density, the deformation and failure modes of the homogeneous steel plate were the same as those of the steel plate or polyurea-coated steel plate, but significantly different from those of the polyurea-coated steel plate. Coating polyurea on the non-blast-facing surface of steel plates exhibited better air blast resistance than coating polyurea on the blast-facing surface. Still, the air blast resistance of the polyurea-coated steel plate was insignificant compared to the homogeneous steel plates of the same weight.

## 1. Introduction

The modern global landscape faces a rapidly evolving crisis, with many regions threatened by warfare and terrorism. Explosive devices produce shock waves, high temperatures, and gases that threaten military and civilian facilities, vehicles, and other targets [1]. As a result, the characteristics and effective protection strategies for warhead explosions under various conditions [2,3,4] have been a key area of research.

To enhance the structure’s anti-blast capability and reduce its damage degree, various methods are employed, such as increasing material thickness, using composite structures, and applying new materials [5,6,7]. Among them, increased material thickness results in greater costs and structural weight, making it challenging to improve blast resistance efficiently. Published studies have shown that using reasonable structures, such as sandwich structures [8,9,10], liquid-filled structures [11,12,13], and resonant structures [14,15,16], can effectively reduce structural damage during explosions. However, structural forms with excellent anti-explosion performance are often complex, thereby limiting their practical use and potentially hindering direct application to strengthen existing protective targets. Hence, applying new materials with better blast resistance to protect the target is a promising reinforcement method, such as using carbon fiber [17,18], glass fiber [19,20], and polyurea [21,22] to strengthen the original structure. Although the optimal explosion-proof material remains unclear, polyurea has received increasing attention due to its excellent performance.

Polyurea is a high-molecular elastomer. It is synthesized from two-component materials and offers a simple and convenient spraying fabrication process, rapid curing rate, excellent resistance to media, and high ductility [23,24]. Moreover, its properties can be flexibly regulated [25], and it adheres well to most materials with strong adhesion [26,27]. Consequently, it has attracted extensive attention to safety protection. In the early stage, polyurea coating was used to reinforce the warhead walls and reduce their fragmentation and collapse under the explosion shock waves [28]. In recent studies, researchers have applied polyurea materials to enhance the anti-blast performance of metal structures [29,30,31], composite materials [20,32], and composite structures [33,34]. Xie et al. [34] investigated that the polyurea/polyaramid composite structure exhibits better anti-blast performance than pure polyaramid at the same surface density. They analyzed that the high temperature generated by the explosion degrades the mechanical properties of the polyurea material. Hence, polyurea must be applied on the non-blast-facing surface to resist the explosion load. Zhang et al. [35] found that polyurea coated on the blast-facing surface tries to separate from the structure as compared to the non-blast-facing surface. This indicated that its anti-blast performance was better than that when it was coated on the blast-facing surface. Jiang et al. [36] found that spraying polyurea on the non-blast-facing surface of the structure enhances the anti-blast capability, effectively reducing the deformation of the structure. Through numerical simulation calculations, they found that the increased thickness of polyurea does not affect the maximum deformed deflection of the structure. Wang et al. [37] investigated that the enhancement effect of polyurea on the anti-blast performance of the structure occurs due to its inhibitory effect on the bending deformation of the structure and the enhanced constraint on the edges of wall components. Zhu et al. [38] conducted experiments and simulation studies on the anti-blast performance of aluminum plates coated with polyurea under repeated explosion loads. Their results showed that polyurea coated on the back surface or both surfaces of the aluminum plate improved its anti-blast performance. Moreover, polyurea significantly reduced the damage to the aluminum plate after the secondary explosion load. Zhang et al. [39] found that the thickness ratio of the structure to polyurea also affected the overall anti-blast performance of the structure. Under the same surface density, reducing the thickness ratio of polyurea to steel substrate reduced the maximum structural deflection.

Based on different scaled blast distances, explosions can be classified as far-field and near-field explosions. Unlike far-field explosions, near-field explosions [40] are mainly characterized by localized load. The localized load gradually transforms into a uniform load as the explosion distance increases [41]. Currently, limited studies have been reported on the dynamic response and failure of polyurea-coated composite structures under near-field explosions. Therefore, further research is needed. In this study, polyurea was coated on the blast-facing and non-blast-facing surfaces of homogeneous steel plate substrates to develop polyurea-coated steel plate composite structures. The failure characteristics of the composite structures under the action of localized air blast shock wave loads were studied. Furthermore, by using the homogeneous steel plate, the influencing laws of blast distance and polyurea coating position on the dynamic response characteristics of the polyurea-coated composite structure were explored.

## 2. Materials and Methods

### 2.1. Experimental Settings

**Figure 1:**
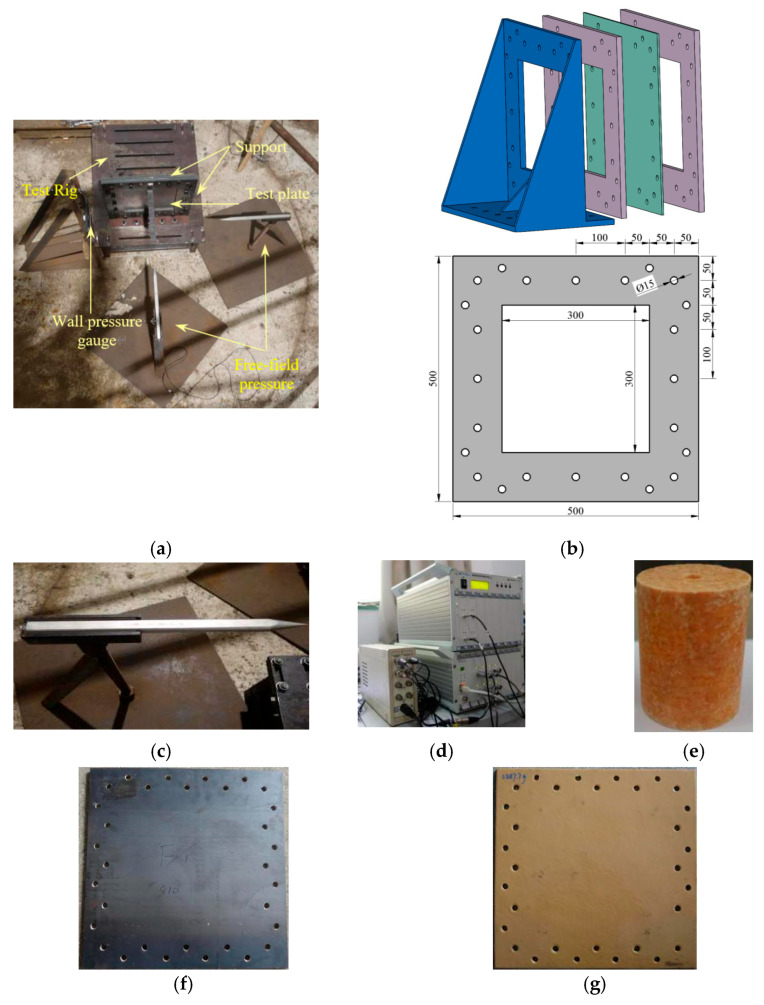
Experimentation: (**a**) the experimental setup; (**b**) the schematic of the experimental plate fixing apparatus; (**c**) free-field pressure gauge and wall pressure gauge; (**d**) dynamic data collector; (**e**) TNT charge; (**f**) bare Q235 steel plate; (**g**) PU-coated steel plate.

Figure 1a presents the overall test layout, the schematic of the target plate fixation device, and the actual experimental instruments and equipment. During the test, the explosive shock wave load was generated by a cylindrical cast TNT with a nominal weight of 200 g. The diameter of the TNT charge column was 50 mm, and its length was 65 mm. A single electric detonator detonated the TNT at the end of the charge column. The bottom surface of the charge column was aligned with the plane of the target plate. The axis of the charge column coincided with the midpoint of the target plate plane. A total of 24 M14 screws and a plain steel frame of 10 mm thickness were used to clamp the composite structure onto a specially designed support of plain steel material, which simulated the fixed boundary conditions. The plane dimensions of the steel frame were 500 × 500 mm^2^, with a square hole of 300 × 300 mm^2^ in the central region, indicating that the actual load-bearing area of the structure was 300 × 300 mm^2^. The support was connected to the fixed platform on the ground through high-strength screws. After the explosion, the platform and the support did not show specific displacement. Moreover, the steel frame and the support did not undergo significant deformation, verifying the stability and reliability of the boundary conditions. Pressure sensors were arranged around the TNT explosive to measure the intensity of the explosion shock waves. Before the test, a grid of 50 × 50 mm^2^ is drawn on the actual load-bearing area of the steel plate to observe its local deformation and display its fracture area clearly. After the explosion test, the target plate was scanned by a three-dimensional (3D) scanner to obtain precise 3D data of the deformed and damaged target plate.

### 2.2. Specimens and Materials

The structural details of the test target plate are given in Table 1. Polyurea of 8 mm in thickness was coated on the blast-facing and non-blast-facing surfaces of two thick steel plates (denoted as S1), which had a thickness of 1.76 mm, and were denoted as 8PU + 1.76S and 1.76S + 8PU, respectively. The surface density was ρA = 21.7 kg/m^2^. To compare the explosion protection efficiencies of different plates, an equal-weight homogeneous steel plate with a thickness of 2.76 mm (denoted as S2) was selected, and its surface density was ρA = 21.6 kg/m^2^.

Q235 steel plate was used with a density of 7850 kg/m^3^, a Poisson’s ratio of 0.3, an elastic modulus of 210 GPa, a yield strength of 235 MPa, a tensile strength of 400–490 MPa, and a ductile failure strain of 22%. The density of the polyurea coating material was 1080 kg/m^3^, with a Shore hardness of 60 ± 1 HD, a tearing strength of 140 kg/cm, a tensile strength of 23.7 MPa, and an elongation rate of 162%.

## 3. Results

To analyze the dynamic response of the target plate under different air blast load intensities, in each test condition, the explosive charge was constant, and only the blast distance was changed. Table 2 lists the specific test conditions and test results. In the table, the blast distance refers to the minimum distance from the bottom surface of the explosive column to the blast-facing surface of the target plate, denoted as DOS. From Table 2, the increased air blast load intensity and changing the target plate result in the deformation and failure modes of the target plate showing completely different characteristics. One test was conducted for each structural configuration. However, the structural damage caused by the standard TNT explosives can be reproduced in the experiment. Therefore, the results obtained in this study are valuable and can be used under similar detonation distances and explosives.

### 3.1. Deformation Modes and Failure Mechanisms

#### 3.1.1. Monolithic Steel Plates

##### 1.76S

**Figure 2:**
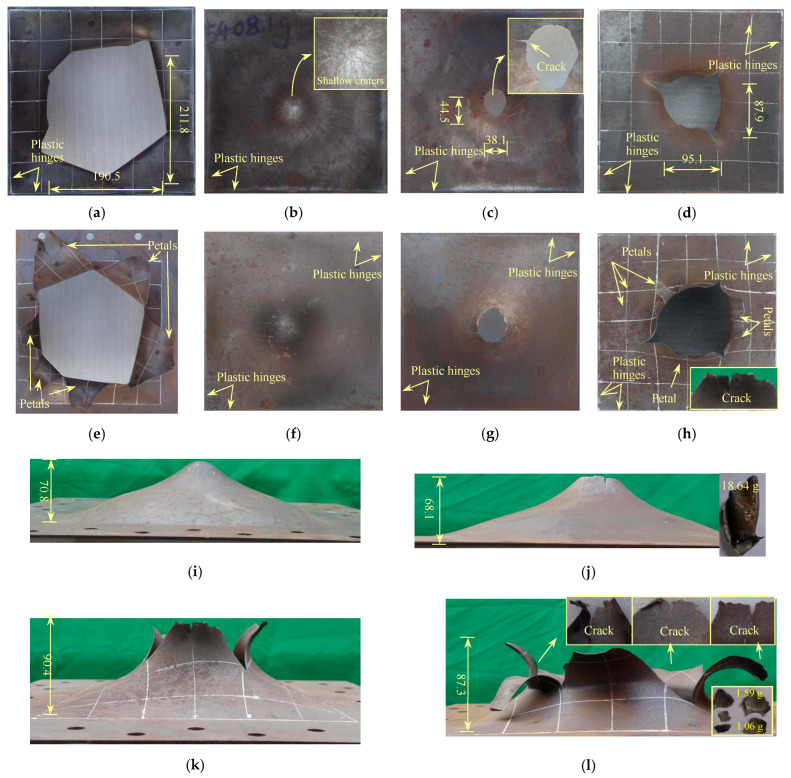
Failure modes of 1.76S and 2.76S plates (mm): (**a**) front view of 1.76S; (**b**) front view of 2.76S when DOS is 90; (**c**) front view of 2.76S when DOS is 75; (**d**) front view of 2.76S when DOS is 60; (**e**) rear view of 1.76S; (**f**) rear view of 2.76S when DOS is 90; (**g**) rear view of 2.76S when DOS is 75; (**h**) rear view of 2.76S when DOS is 60; (**i**) Side view of 2.76S when DOS is 90; (**j**) side view of 2.76S when DOS is 75; (**k**) side view of 2.76S when DOS is 60; (**l**) side view of 1.76S when DOS is 90.

Figure 2 shows the deformation and failure morphologies of the 1.76S and 2.76S target plates. When DOS is 90 mm, the failure mode of the 1.76S target plate is flexural deformation, plugging breach, plus petal-like cracking. An irregular, large breach with a maximum size of about 211.8 mm is formed at the central region of the target plate. The overall mass of the target plate decreases by 27.5 g, from which it can be estimated that the average diameter of the plugging block is about 50.2 mm. The number of petals on the non-blast-facing surface is six, with the maximum protrusion height of about 87.3 mm. There are plastic twisted lines at the base of the petals, and each petal has a rotation angle exceeding 90°. The front edge thickness of the petal is reduced due to the tensile fracture effect. Moreover, cracks can be observed on the front edge of the large petals. The front edge of the small petals is relatively flat with no cracks. From the collected fragments of the 1.76S target plate, the fragments are incomplete and broken into small debris. The masses of the three larger pieces are about 1.59 g, 1.06 g, and 0.92 g. The fragments showed a particular curvature, indicating that the material in the failure zone does not form a complete group of plugging blocks but fractures into small pieces during the deformation process.

##### 2.76S

As the thickness of the homogeneous plate increases, its anti-blast capability improves significantly. When DOS is 90 mm, the failure mode of the 2.76S target plate is overall large flexural deformation, and the maximum deflection is 68.1 mm. A few shallow pits in the central zone of the blast-facing surface are caused by the high-speed impacts of partly detonated explosive residue. Under the tensile effect, the thickness of the target plate gradually decreases from the plate edge to the position with the maximum deformed deflection, and the thinnest point is 2.09 mm, with a reduction rate of 24.3%. Based on the bidirectional strain assumption, the strain is observed in the plate thickness as *ε*_h_ = *h*_1_/*h* − 0.243, the strain along with the planar direction as $\varepsilon_{x}=\varepsilon_{y}=\sqrt{h/h_{1}}-1=0.149$, and the effective plastic strain as $\varepsilon_{eq}=\sqrt{2\left[{\left(\varepsilon_{1}-\varepsilon_{2}\right)}^{2}+{\left(\varepsilon_{2}-\varepsilon_{3}\right)}^{2}+{\left(\varepsilon_{3}-\varepsilon_{1}\right)}^{2}\right]/9}=2(\varepsilon_{x}-\varepsilon_{h})/3=0.261$. The components of deformation energy absorbed in the 2.76S steel plate are also calculated: the bending deformation energy as *U*_b_ = 0.43 kJ (2.01%), the tensile deformation energy as *U*_e_ = 20.67 kJ (96.78%), and the plastic hinge energy as *U*_h_ = 0.26 kJ (1.21%). Therefore, in the failure mode of large flexural deformation, the 2.76S steel plate dissipates most of the energy through severe tensile deformation, followed by the bending deformation. The plastic hinge energy absorption accounts for the least proportion.

When DOS is 75 mm, the fracture mode of the target plate is flexural deformation plus plugging breach. The plastic twisted lines at the edge of the target plate are more noticeable compared to those of DOS, which is 90 mm. A circular breach with an average diameter of about 41.3 mm is formed at the central region of the target plate, and the thickness at the edge of the fracture is about 1.60 mm (with a reduction rate of 42.0%). The maximum protrusion height on the non-blast-facing surface of the target plate is about 70.8 mm. When DOS is 60 mm, the target plate is severely damaged, and the failure mode exhibits flexural deformation with plugging breach and petal-like cracking. The target plate still undergoes a large flexural deformation, and a significant plugging breach is formed at the central region of the target plate, with a maximum size of about 95.1 mm. The target plate has a total of 6 petals, with their flipping angles all over 90°, and the maximum protrusion height is about 90.4 mm. The petal size distribution is uneven, and two noticeable cracks can be observed on the leading edge of the largest-sized petal. The overall mass loss of the target plate is 24.6 g, and the estimated average diameter of the plugging block is about 37.7 mm.

#### 3.1.2. 1.76S + 8PU Plates

**Figure 3:**
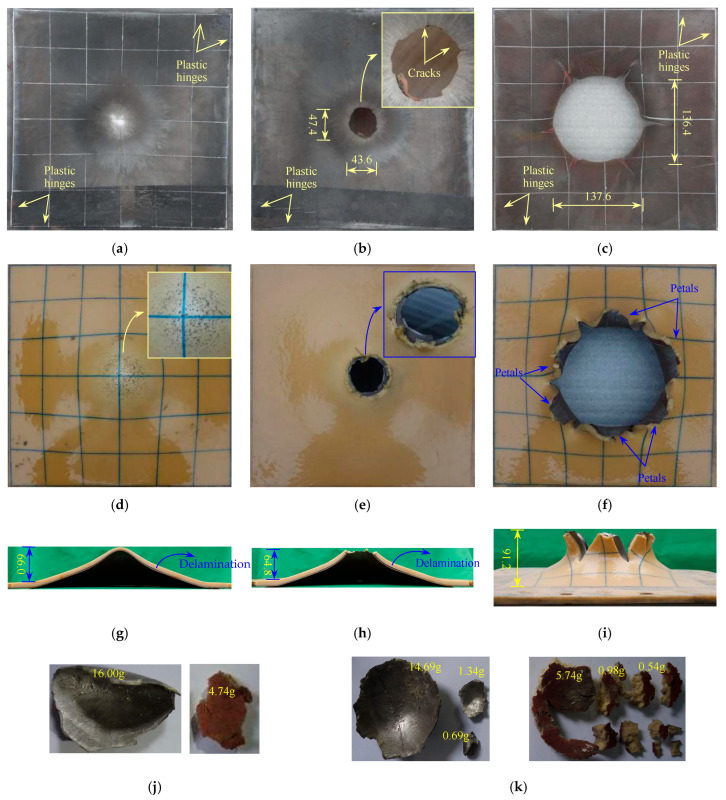
Failure modes of polyurea r and steel plate layers for 1.76S + 8PU configurations (mm): (**a**) front view when DOS is 75; (**b**) front view when DOS is 75; (**c**) front view when DOS is 60; (**d**) rear view when DOS is 90; (**e**) rear view when DOS is 75; (**f**) rear view when DOS is 60; (**g**) cross-sectional view when DOS is 90; (**h**) cross-sectional view when DOS is 75; (**i**) side view when DOS is 60; (**j**) structure fragments when DOS is 75; (**k**) structure fragments when DOS = 60.

Figure 3 shows the deformation and failure morphology of the 1.76S + 8PU target plate. When DOS is 90 mm, the deformation and failure mode of the 1.76S + 8PU target plate are overall large flexural deformations. The failure morphology of the blast-facing surface is roughly the same as that of the 2.76S target plate. Many small, shallow circular pits can be observed near the maximum protrusion position of the polyurea coating surface on the non-blast-facing surface. In contrast, the surface remains smooth in the region without large deformation. The maximum deflection of the blast and nonblast facing surfaces of the target plate is about 65.5 mm and 66.0 mm, respectively. The diameter where the polyurea layer detaches from the substrate is about 188 mm. At the maximum deformation point of the target plate, although the polyurea layer detaches from the substrate, it still closely adheres to the substrate. The polyurea coating undergoes a thickness reduction and severe tensile deformation, and the thinnest point is about 4.45 mm, with a reduction rate of 44.4%.

When DOS is 75 mm, the deformation and failure mode of the target plate is large flexural deformation plus plugging breach, and the failure morphology of the blast-facing surface is the same as that of the 2.76S target plate. The maximum protrusion height of the non-blast-facing surface of the target plate is about 64.8 mm. The average diameter of the plugging breach at the central region is about 45.5 mm, with unsmooth fracture edges. The overall mass loss of the target plate is 24.7 g, from which it can be estimated that the average diameter of the plugging block is about 37.4 mm. The diameter where the steel substrate plate and the polyurethane coating are delaminated is about 225 mm. From the collected structural fragments, a group of plugging blocks weighing 16.02 g is produced from the central region of the substrate plate. In contrast, the polyurethane layer is fractured, with the largest fragment weighing 4.72 g. The collected plugging blocks of the substrate plate show a specific curvature due to the plate undergoing significant bulging before the formation of the plugging block. The surface of the polyurethane fragment is smooth, indicating that the high toughness property of the polyurethane material causes it to rebound after the fracture. On the other hand, this also suggests that the dynamic strength of the polyurethane material is poor, and it is prone to fracture when subjected to high loading speeds.

When DOS is 60 mm, the fracture mode of the target plate is flexural deformation, plugging breach, and petal-like cracking. There is a large breach in the central region of the target plate, with a diameter of about 137.1 mm. A total of 6 petal-like cracks are produced with flipping angles over 90°. The maximum protrusion height is about 91.2 mm. The size distribution of the petal bodies is relatively uniform, and an obvious thickness reduction is observed at the edges. The positions of the petal bodies of the polyurethane layer are coordinated with those of the steel plate. The overall mass loss of the target plate is 26.3 g, and the average diameter of the plugging block is estimated to be 39.3 mm. From the collected structural fragments, a group of plugging blocks (weighing 14.68 g) is produced in the central region of the substrate plate. In contrast, the polyurethane layer is fractured, with the largest fragment weighing 5.74 g.

#### 3.1.3. 8PU + 1.76S Plates

**Figure 4:**
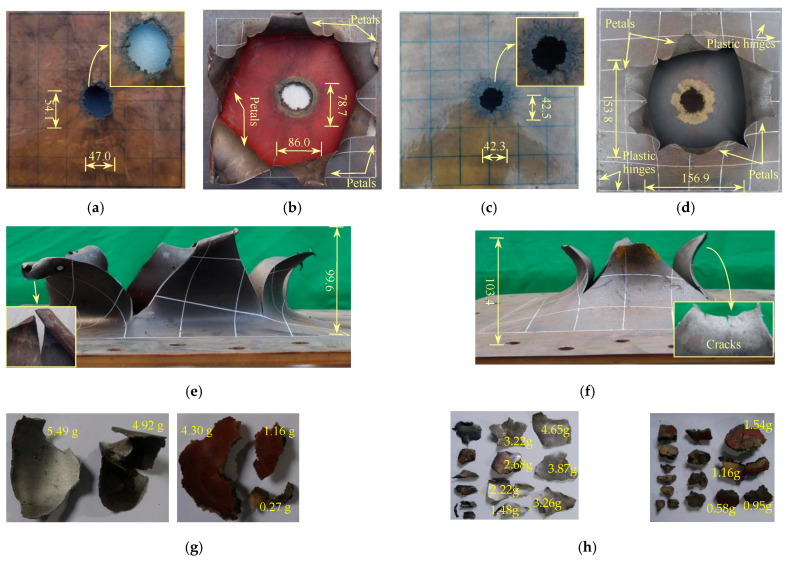
Failure modes of polyurea and steel plate layers for 8PU + 1.76S configuration (mm): (**a**) front view when DOS is 90; (**b**) rear view when DOS is 90; (**c**) front view when DOS is 75; (**d**) rear view when DOS is 75; (**e**) side view when DOS is 90; (**f**) side view when DOS is 75; (**g**) structure fragments when DOS is 90; (**h**) structure fragments when DOS is 75.

Figure 4 shows the deformation and failure morphology of the 8PU + 1.76S target plate. When DOS is 90 mm, the blast-facing surface of the 8PU + 1.76S plates remains flat, and a circular plugging hole with a diameter of about 50.5 mm is formed in the central region. The edge of the hole is not smooth. The fracture mode of the steel substrate plate is flexural deformation with plugging breach and petal-like cracking. Some twisted plastic lines are observed at the edges. The number of petals is 6, and the maximum protrusion height of the petal body is about 99.6 mm. The cracks on both sides of the petal body are relatively long, extending to the plate edges. The front end of the petal body is relatively flat, and a obvious crack exists at the centre of the largest petal body. The overall mass of the target plate decreases by 46.9 g, from which it can be estimated that the average diameter of the plugging block is about 52.0 mm. The steel substrate plate and the polyurethane coating are entirely separated. The collected steel plate structural fragments are found to be incompletely fractured. The masses of the two largest fragments are 5.49 g and 4.92 g, respectively, and both fragments have a certain curvature. In contrast, the polyurethane coating is severely fractured, and the masses of the two largest fragments are 4.30 and 1.16 g, respectively.

When DOS is 75 mm, the failure mode of the target plate is like that of the target plate in Test 3. The polyurea coating remains flat, and a hole with a diameter of about 39.0 mm is formed at the central region. The surrounding area of the hole is black, which may be caused by the elevated temperature during the detonation. The fracture mode of the steel substrate plate is flexural deformation with plugging breach and petal-like cracking. There are five petals, and the maximum protrusion height of the petals is about 103.4 mm. The thickness of the front edge of the petals is significantly reduced. Apparent cracks can be observed on the front edge of the large petals. In contrast, the front edge of the small petals is relatively flat, with no cracks. The maximum size of the breach is about 156.9 mm. The overall mass of the target plate decreases by 24.8 g, from which it can be estimated that the average diameter of the initial plugging zone of the steel substrate plate is about 38.1 mm. The steel substrate plate and the polyurea coating are entirely separated. From the collected structural fragments, both the substrate plate and the polyurea coating are severely fragmented. The masses of some larger substrate plate fragments are 4.65 g, 3.87 g, 3.26 g, 3.22 g, 2.68 g, 2.22 g, and 1.48 g, while those of some larger polyurea coating fragments are 1.54 g, 1.16 g, 0.95 g, and 0.58 g. The obstruction of the support on the motion of the front end of the petal body restricts the flipping of the petal body, resulting in insufficient development of the petal body.

**Figure 5:**
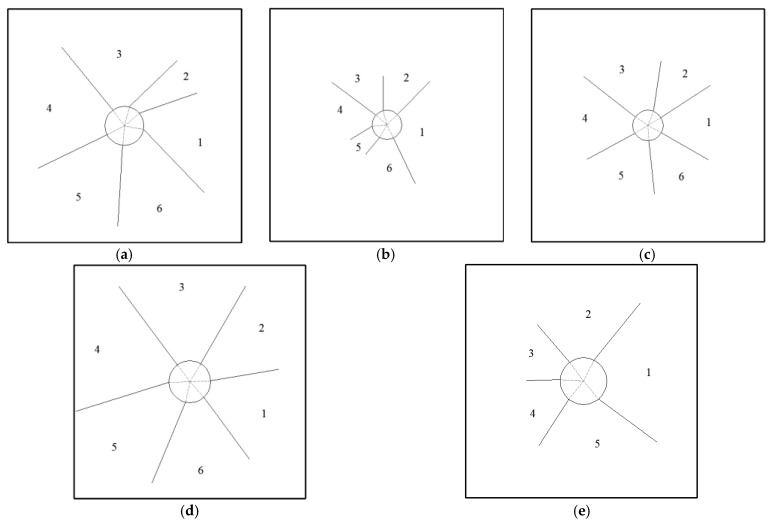
The crack propagation paths of ruptured steel base plate: (**a**) 1.76S when DOS is 90; (**b**) 2.76S when DOS is 60; (**c**) 1.76S + 8PU when DOS is 60; (**d**) 8PU + 1.76S when DOS is 90; (**e**) 8PU + 1.76S when DOS is 75.

**Figure 6:**
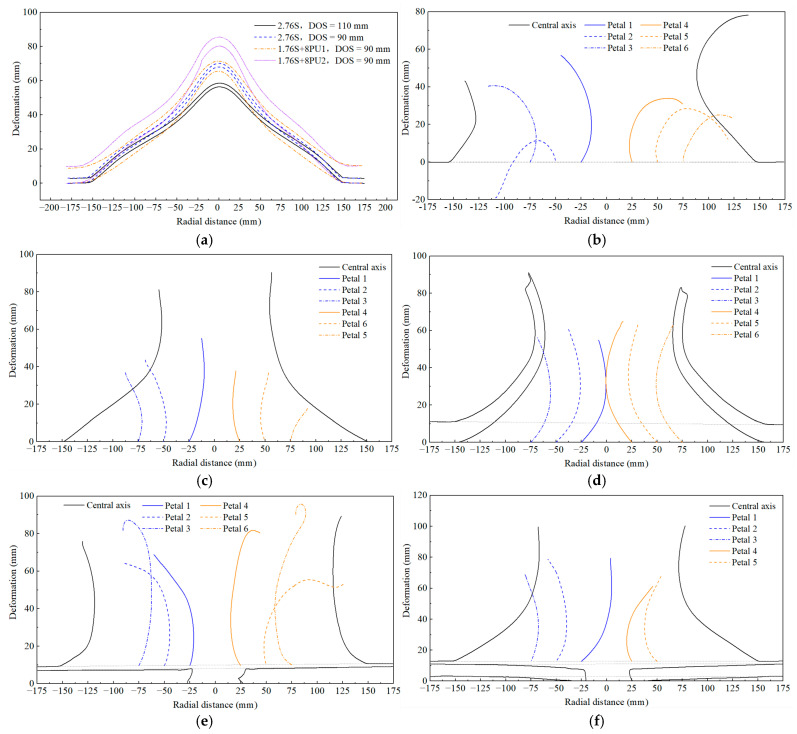
Deformation profiles of steel base plate and petals of ruptured steel base plate: (**a**) 2.76S, 1.76S+ 8PU; (**b**) 1.76S when DOS is 90; (**c**) 2.76S when DOS is 60; (**d**) 1.76S+ 8PU when DOS is 60; (**e**) 8PU + 1.76S when DOS is 90; (**f**) 8PU + 1.76S when DOS is 75.

Based on the above images, the crack propagation paths of ruptured steel base plate in differet tests are given in Figure 5. And Figure 6 shows deformation profiles of steel base plate and petals of ruptured steel base plate. Overall, under the localized air blast loads, the polyurea-coated steel plate exhibits three failure modes: overall flexural large deformation, overall flexural deformation plus plugging breach, and overall flexural deformation with plugging breach and petal-like cracking. Under the same blast distance and surface density, the deformation failure modes and failure degrees of the homogeneous steel plate are the same as those of the steel plate/polyurea structure, but significantly different from those of the polyurea/steel plate structure.

### 3.2. Air Blast Resistance and Deformation of Polyurea-Coated Steel Plates

#### 3.2.1. Comparison of Air Blast Resistance

In this study, the degree of structural deformation and failure is taken as the evaluation index for air blast resistance. When DOS is 90 mm, the 8PU + 1.76S structure suffered the most severe damage, followed by 1.76S, 1.76S + 8PU, and 2.76S, which were comparable and demonstrated the lightest degree of damage. When DOS is 75 mm, the 8PU + 1.76S structure suffered the most severe damage, with 1.76S + 8PU and 2.76S showing lower levels of damage than 8PU + 1.76S. When DOS = 60 mm, the failure degrees of 2.76S and 1.76S + 8PU are comparable. Spraying the polyurea coating on the non-blast-facing surface can significantly improve the anti-blast capability of the polyurea-coated steel plate. Under the localized air blast loads, the failure degree of the 1.76S + 8PU does not show a significant improvement compared to that of the 2.76S homogeneous steel plate with the same mass. Therefore, the air blast resistance of the polyurea-coated steel plate is not promising over that of the homogeneous steel plate with the same mass. Specifically, when DOS is 90 mm, both structures 1.76S + 8PU and 2.76S exhibit significant overall flexural deformation, with deformation sizes of 65.5 and 68.1 mm, indicating that the former has a maximum deformation of 3.82% less than the latter. When DOS is 75 mm, both structures 1.76S + 8PU and 2.76S produced plugging breaches with maximum diameters of 47.4 and 44.5 mm, respectively. At this point, 1.76S + 8PU suffered slightly more damage than 2.76S. When DOS is 60 mm, both structures 1.76S + 8PU and 2.76S produced plugging breach plus petal-like cracking with maximum diameters of 95.1 and 137.6 mm, respectively. This implies that the polyurea increased the damage suffered by the steel plate in the 1.76S + 8PU structure at this time. This indicates that the steel/polyurea structure may not be suitable for protecting near-field explosions.

#### 3.2.2. Failure Process

**Figure 7:**
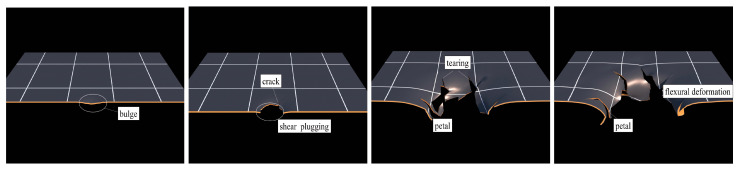
The failure process of steel/polyurea composite structures.

**Figure 8:**
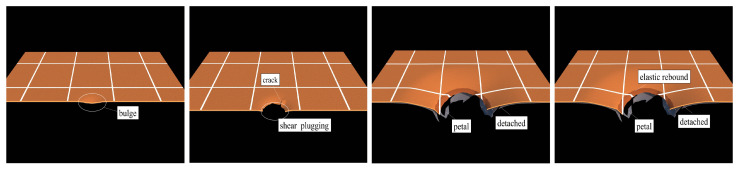
The failure process of the polyurea/steel composite structures.

The failure process of differet structure configuration are given in Figure 7 and Figure 8. Under the localized blast shock wave, the central zone of the polyurea-coated steel plate undergoes localized bulging, and the bulging speed in the local bulging zone is exceptionally high. Due to the large speed gradient, shearing tensile failure occurs around the local bulging zone, producing a plugging breach. Under the shock waves, the polyurea-coated steel plate undergoes an overall bending deformation, and the small cracks around the plugging breaches initiate extended cracks. The cracks propagate, resulting in petal-like cracking failure. When the polyurea coating is applied to the blast-facing surface (8PU + 1.76S plate), due to the incoordination of the deformation of the polyurea layer and the steel plate, the interface between the polyurea coating and the steel plate constantly detaches during the overall flexural deformation and petal-like cracking failure of the steel plate. The polyurea layer no longer deforms in coordination with the steel plate to induce petal-like cracking failure, but undergoes elastic rebound. When the polyurea coating is applied to the non-blast-facing surface (1.76S + 8PU plate), the polyurea and the steel plate substrate continuously deform in coordination, and the interface detachment is infrequent. The steel plate pushes the polyurea to the failure mode of overall flexural deformation with petal-like cracking.

### 3.3. Analysis of Stress Wave Propagation Process

The elastic longitudinal wave velocities of the steel substrate plate (*C*_s_) and polyurethane coating (*C*_pu_) are 5172.2 m/s and 461.5 m/s, respectively. The wave impedance ratio (*n*_s-pu_) between the two materials is 81.5, the reflection coefficient (*F*_s-pu_) is −0.98, and the transmission coefficient (*T*_s-pu_) is 0.024. In the 8PU + 1.76S composite structure, the shock wave first interacts with the polyurethane coating and generates a certain intensity of compression wave, which is assumed to be *σ*_pu_. Then the reflected stress at the interface between the polyurethane coating and the substrate (*σ*′_pu_) and the transmitted stress (*σ*′_s_) in the substrate are 0.98*σ*_pu_ and 1.98*σ*_pu_, respectively. The intensity of the reflected compression wave is high, while the intensity of the transmitted compression wave (the incident stress wave in the substrate) is nearly doubled. In the 1.76S + 8PU composite structure, the shock wave encounters the steel substrate, and the intensity of the generated compression wave is assumed to be *σ*_s_. The intensity of the reflected stress wave at the interface between the substrate and the polyurethane coating is comparable to that of the tensile wave (*σ*′_s_ is −0.98*σ*_s_). In contrast, the intensity of the transmitted compression wave significantly attenuates (*σ*′_pu_ is 0.024*σ*_s_).

**Figure 9:**
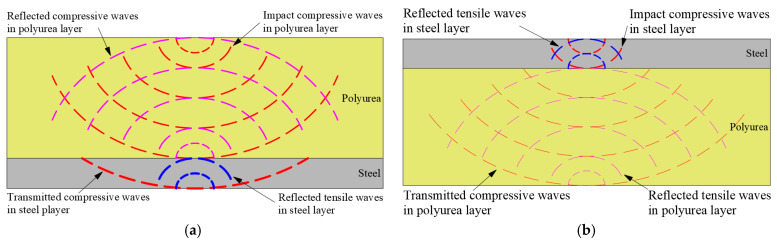
Schematic diagram of stress wave propagation in polyurea-coated steel plate composite structures: (**a**) 8PU + 1.76S; (**b**) 1.76S + 8PU.

Hence, as the Figure 9 shows, when the polyurea coating is applied to the blast-facing surface of the steel plate, the discontinuity in wave impedance between the polyurea coating and the steel plate amplifies the intensity of the shock wave. Although the polyurea material exhibits good toughness, its strength is low. Under the strong shock waves, the fast-loading speed causes the central region of the polyurea material to experience premature plugging-type failure. As a result, the non-blast-facing surface of the steel plate is unrestrained and its motion is not restricted, leading to severe damage. When the polyurea coating is applied to the non-blast-facing surface of the steel plate, the severe mismatch in wave impedance between the polyurea coating and the steel plate dissipates energy and blocks the propagation of stress waves. Moreover, during the coordinated deformation process between the polyurea coating and the steel plate, the polyurea material undergoes continuous glass transition, which not only dissipates energy but also enhances the material’s stiffness. Thus, the bending resistance of the steel plate increases, and the damage resistance performance of the polyurea-coated steel plate improves.

## 4. Conclusions

This experimental study focused on the deformation and failure of homogeneous steel plates and polyurea-coated steel plates under the localized air blast loads. The study investigated the effects of blast distance and polyurethane coating position on the deformation and failure modes of polyurea-coated steel plate composite structures. It clarified the dynamic response failure and stress wave propagation characteristics of the polyurea-coated steel plates. The main conclusions are as follows:

(1) Under the localized air blast loads, with the decreased blast distance, the steel plate/polyurea coating composite structures experienced three different deformation failure modes, i.e., the overall large flexural deformation, overall flexural deformation with plugging breach, and overall flexural deformation with plugging breach and petal-like cracking. In contrast, the failure mode of the polyurea/steel plate composite structure resulted in the entire detachment of the polyurea layer and the steel substrate. The polyurea layer was overall flat with local plugging breaches, and the steel substrate showed the fracture mode of flexural deformation with plugging breaches and petal-like cracking.

(2) Under the same blast distance and surface density, the deformation and failure mode and deformation degree of the steel plate/polyurea composite structure were the same as those of the homogeneous steel plate. In contrast, these were significantly different from those of the polyurea/steel plate composite structure. Specifically, when DOS is 90 mm, both structures 1.76S + 8PU and 2.76S exhibit significant overall flexural deformation, with the former having a maximum deformation of 3.82% less than the latter. When the DOS is 75 mm, both structures 1.76S + 8PU and 2.76S produced plugging breaches with maximum diameters of 47.4 mm and 44.5 mm, respectively. When DOS is 60 mm, both structures 1.76S + 8PU and 2.76S produced plugging breach plus petal-like cracking with maximum diameters of 95.1 mm and 137.6 mm, respectively.

(3) Under the localized air blast loads, a steel plate with polyurea coating on the rear surface can significantly improve the protective ability of the steel plate compared to the original steel structure. However, when coated on its front surface, the degree of damage to the original steel structure increases. Compared to the steel structures with equal surface density, the steel/polyurea structure showed a minor deformation when the blasting distance was far. Still, as the blasting distance decreased, the rupture size of the steel/polyurea structure increased.

## Figures and Tables

**Table 1:** Structural details of the test target.

Configuration	Geometry	Layer Arrangement, Thickness (mm)	ρ_A_ (kg/m^2^)
1.76S	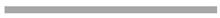	S, 1.76	13.7
2.76S	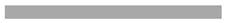	S, 2.76	21.6
1.76S + 8PU	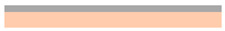	S + PU, 1.76 + 8	21.7
8PU + 1.76S	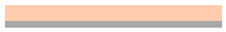	PU + S, 8 + 1.76	21.7

**Table 2:** Various test configurations and blast experimental results.

Test Conditions	Structure Configuration	DOS (mm)	Deformation and Failure Modes of the Target Plate
Test 1	1.76S	90	Flexural deformation plus plugging breach plus petal-like cracking.
Test 2	2.76S		Overall, large flexural deformation without cracks.
Test 3	8PU + 1.76S		Plugging breach at the central region of polyurea coating, flexural deformation, plugging breach, plus petal-like cracking of the substrate plate
Test 4	1.76S + 8PU		Overall, large flexural deformation without cracks.
Test 5	2.76S	75	Overall, there is a large flexural deformation, with a plugging breach at the central region.
Test 6	1.76S + 8PU		Overall, there is a large flexural deformation, with a plugging breach at the central region.
Test 7	8PU + 1.76S		Plugging breach at the central region of polyurea coating, Flexural deformation, plugging breach, plus petal-like cracking of the substrate plate.
Test 8	2.76S	60	Flexural deformation plus plugging breach plus petal-like cracking.
Test 9	1.76S + 8PU		Flexural deformation plus plugging breach plus petal-like cracking.

## Data Availability

The original contributions presented in the study are included in the article; further inquiries can be directed to the corresponding author.

## Dual-Use Research of Concern Statement

Our research is limited to analyzing the influence of polyurea on the deformation and failure modes of steel plates under blast loading, and does not involve the study of explosives. We believe our findings contribute to mitigating blast damage to structures such as buildings and vehicles, and do not pose a threat to public health or national security. The authors acknowledge the dual use potential of the research involving blast and confirm that all necessary precautions have been taken to prevent potential misuse. As an ethical responsibility, authors strictly adhere to relevant national and international laws about DURC. Authors advocate for responsible deployment, ethical considerations, regulatory compliance, and transparent reporting to mitigate misuse risks and foster beneficial outcomes.
